# Quantitative Proteomics Reveal Distinct Protein Regulations Caused by *Aggregatibacter actinomycetemcomitans* within Subgingival Biofilms

**DOI:** 10.1371/journal.pone.0119222

**Published:** 2015-03-10

**Authors:** Kai Bao, Nagihan Bostanci, Nathalie Selevsek, Thomas Thurnheer, Georgios N. Belibasakis

**Affiliations:** 1 Oral Translational Research, Institute for Oral Biology, Center of Dental Medicine, University of Zurich, Zurich, Switzerland; 2 Functional Genomics Center Zurich, University of Zurich, Zurich, Switzerland; 3 Oral Microbiology and Immunology, Institute for Oral Biology, Center of Dental Medicine, University of Zurich, Zurich, Switzerland; University of Florida, UNITED STATES

## Abstract

Periodontitis is an infectious disease that causes the inflammatory destruction of the tooth-supporting (periodontal) tissues, caused by polymicrobial biofilm communities growing on the tooth surface. Aggressive periodontitis is strongly associated with the presence of *Aggregatibacter actinomycetemcomitans* in the subgingival biofilms. Nevertheless, whether and how *A*. *actinomycetemcomitans* orchestrates molecular changes within the biofilm is unclear. The aim of this work was to decipher the interactions between *A*. *actinomycetemcomitans* and other bacterial species in a multi-species biofilm using proteomic analysis. An *in vitro* 10-species “subgingival” biofilm model, or its derivative that included additionally *A*. *actinomycetemcomitans*, were anaerobically cultivated on hydroxyapatite discs for 64 h. When present, *A*. *actinomycetemcomitans* formed dense intra-species clumps within the biofilm mass, and did not affect the numbers of the other species in the biofilm. Liquid chromatography-tandem mass spectrometry was used to identify the proteomic content of the biofilm lysate. A total of 3225 and 3352 proteins were identified in the biofilm, in presence or absence of *A*. *actinomycetemcomitans*, respectively. Label-free quantitative proteomics revealed that 483 out of the 728 quantified bacterial proteins (excluding those of *A*. *actinomycetemcomitans*) were accordingly regulated. Interestingly, all quantified proteins from *Prevotella intermedia* were up-regulated, and most quantified proteins from *Campylobacter rectus*, *Streptococcus anginosus*, and *Porphyromonas gingivalis* were down-regulated in presence of *A*. *actinomycetemcomitans*. Enrichment of Gene Ontology pathway analysis showed that the regulated groups of proteins were responsible primarily for changes in the metabolic rate, the ferric iron-binding, and the 5S RNA binding capacities, on the universal biofilm level. While the presence of *A*. *actinomycetemcomitans* did not affect the numeric composition or absolute protein numbers of the other biofilm species, it caused qualitative changes in their overall protein expression profile. These molecular shifts within the biofilm warrant further investigation on their potential impact on its virulence properties, and association with periodontal pathogenesis.

## Introduction

Oral biofilms play an important role in periodontal disease [1], a primary reason for human adult tooth loss [2]. With more than 700 species identified in the oral cavity [3], this biofilm presents a complex and dynamic ecosystem, whose growth is dictated by microenvironmental factors. As proof of concept, studies in murine models have demonstrated the multiple species biofilms display increased pathogenicity [4,5], reflecting the increased alveolar bone loss [6–8], which is the hallmark of periodontitis. Within a biofilm, the bacteria exert a significantly increased virulence and resistance to the host immune defences. Therefore, “traditional” experimental models that simply study single individual bacterial species might not be optimal to acknowledge the role of oral biofilms in periodontal diseases.

To understand the role of the oral biofilms in disease, it is necessary to unravel the relationships between their constituent species. Based on co-aggregation experiments, it is estimated that there can be numerous interactions between various microbial species of the human oral cavity [9]. Such aggregations reflect the formation of biofilms, both by defining the early colonizing events of the tooth surfaces, and generating optimal microenvironments for the later colonizing species [10]. Interestingly, in a multispecies biofilm model similar to the one employed in this study, it is shown that in the absence of the “early colonizing” species, the “late colonizing” species form different structures within the biofilm [11]. A number of experimental studies were also performed to investigate the detailed effects of virulence factors of one species to another, within a multi-species biofilm community. For example, BspA protein from *Tannerella forsythia* favours the co-aggregation with *Fusobacterium nucleatum* [12], whereas the lysine and arginine gingipains of *Porphyromonas gingivalis* regulated the growth of *T*. *forsythia* [13] and of *Treponema denticola* [13,14], respectively; *Aggregatibacter actinomycetemcomitans* utilizes L-lactate from *Streptococcus gordonii* as energy source [15]. However, not all relationships within biofilms are synergistic. For example, streptococcal arginine deiminase inhibits the expression of fimA from *Porphyromonas gingivalis* and thus abrogates colonization [16]; AI-2 of *A*. *actinomycetemcomitans* inhibits biofilm formation of *Candida albicans* [17]. Still, most of the models used to investigate inter-species associations involve pair-wise bacterial comparisons, and the obtained data might be an oversimplified version of the reality. Using multi-species biofilm models may be closer to the *in vivo* situation, and may allow for the extrapolation of biological data that is more clinically relevant. In recent years, a 10-species *in vitro* “subgingival” biofilm model has been established and optimized in order to address such issues [11,18–22]. In the present study, this model was evolved further to incorporate *A*. *actinomycetemcomitans*, a highly leukotoxic species that is strongly associated with aggressive forms of periodontitis occurring among young individuals [23]. The many virulence factors of *A*. *actinomycetemcomitans* identified are its putative “weapons” against the host immune armament, including polymorphonuclear leukocytes, T-lymphocytes and macrophages [24]. In a biofilm environment, these functions may not only be favouring *A*. *actinomycetemcomitans* itself, but might also support all other species in the biofilm in escaping the host immune system. Besides, this bacterium affects other species commonly found in subgingival biofilms, including *P*. *gingivalis* [25] and *F*. *nucleatum* [26]. As suggested by Hajishengallis et al [27,28], a keystone pathogen for periodontal infection might not actually be the dominant species within the biofilm, but may induce changes in other constituent species. Deciphering the protein regulations across the biofilm could therefore be crucial in understanding the role of the individual species in the integrity and function of the biofilm. Yet, most studies have addressed the role of only one or a handful of proteins, rather than the overall protein profile in a biofilm.

Proteomics provide an important novel approach to extract detailed information of cellular regulatory mechanisms on the protein level at a large scale. With the utilization of mass spectrometry-based technologies, it is possible to identify and quantify thousands of proteins from complex biological samples in one run [29–32]. In a biofilm environment, this tool could not only support the identification of regulatory proteins, but also evaluate the trend of their regulation at a universal level. Based on the above considerations, a label-free quantitative proteomic approach was employed to quantify the protein expressions and cluster their functions, in an 11-species *in vitro* “subgingival” biofilm, or its 10-species variant lacking *A*. *actinomycetemcomitans*. Hence, by inference, the relative regulatory roles of *A*. *actinomycetemcomitans* in the biofilm were deduced.

## Results

### *Aggregatibacter actinomycetemcomitans* forms dense clusters without altering the composition of the other species alters in the biofilm

**Fig 1:**
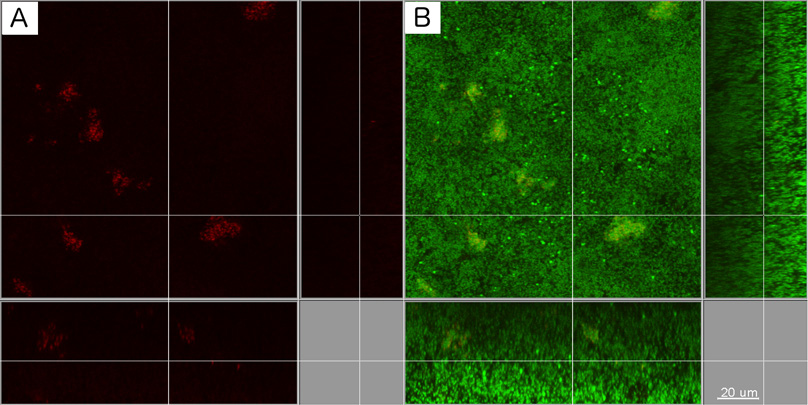
Localization of *A*. *actinomycetemcomitans* within the biofilms. (A) *A*. *actinomycetemcomitans* cells were stained by fluorescence *in situ* hybridization using Cy3-labelled 16 S rRNA oligonucleotide probe Act 639 (red), (B) *A*. *actinomycetemcomitans* cells (red) and rest of the biofilm cells was counter stained with a mixture of YoPro-1 iodide and Sytox Green (green). Scale bar length: 20 μm.

Upon completion formation after 64 h, the biofilms were either kept intact for the confocal laser scanning microscopy (CLSM) analysis or harvested in suspensions for further quantification. The numbers for each individual species within the 11-species biofilm with 10-species biofilm (with or without *A*. *actinomycetemcomitans*, respectively) were quantified using quantitative real-time polymerase chain reaction (qPCR) (Table 1). Interestingly, the t-tests indicated that there were no significant (*P*<0.01) differences in the numbers of each individual bacterial species within the biofilms, irrespective of the presence or absence of *A*. *actinomycetemcomitans*. To further study the localization of *A*. *actinomycetemcomitans* within the biofilm, the biofilm structure was investigated by CLSM (Fig. 1). It was demonstrated that *A*. *actinomycetemcomitans* formed dense clumps, or aggregates, with its own species.

**Table 1:** Quantitative composition of the 11-species or 10-species biofilm after 64 h cultivation.

	11-species biofilm		10-species biofilm	
Species	Mean^1^	SD	Mean^1^	SD
*A*. *actinomycetemcomitans*	3.76E+08	1.17E+08	-	-
*A*. *oris*	2.37E+08	3.99E+07	1.01E+08	4.10E+07
*F*. *nucleatum*	9.04E+09	5.11E+08	6.02E+09	3.20E+09
*C*. *rectus*	9.14E+08	9.36E+07	1.55E+09	8.07E+08
*P*. *gingivalis*	8.14E+07	2.40E+07	3.25E+08	3.44E+08
*P*. *intermedia*	5.58E+09	1.72E+09	1.22E+09	8.48E+08
*S*. *anginosus*	1.66E+09	4.45E+08	1.88E+09	1.28E+09
*S*. *oralis*	4.22E+09	4.19E+08	2.60E+09	1.35E+09
*T*. *denticola*	3.96E+07	1.03E+07	3.91E+07	2.73E+07
*T*. *forsythia*	5.64E+06	3.37E+06	3.71E+06	3.95E+06
*V*. *dispar*	8.20E+09	9.96E+08	3.64E+09	1.69E+09

1:Quantification was performed used qPCR for each species. The data is expressed as the bacterial mean counts ± standard deviation (SD) from triplicate biofilm cultures. No statistical differences (P ≤ 0.01) of between the groups were found within the same species by student t-test.

### *Aggregatibacter actinomycetemcomitans* causes shifts in the numbers of others species’ detectable proteins in the biofilm

**Fig 2:**
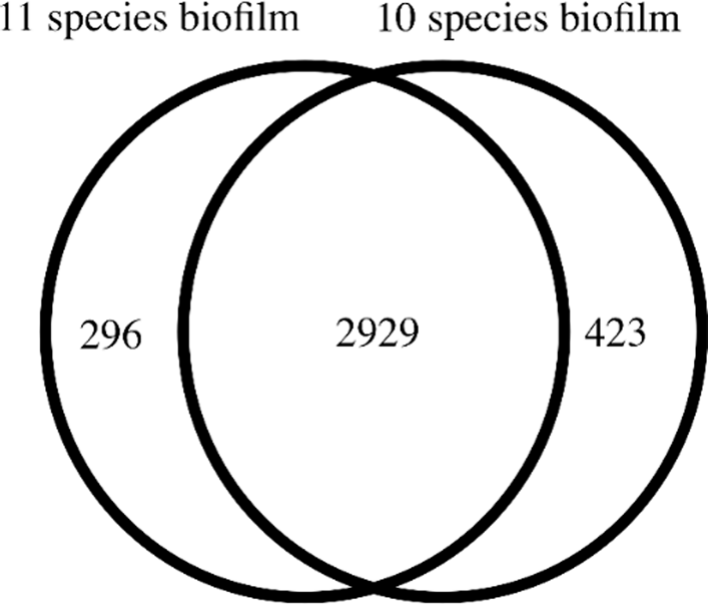
Venn diagram of identified proteins and their overlapping groups between the 11-species and 10-species biofilm. Protein numbers for each category were listed. Details of proteins in each group were listed in S1 Table.

Total protein was extracted from biofilm suspensions for global proteomic characterization of the differences between the two forms of biofilms, each with biological triplicates. It was found that a total of 3225 and 3352 proteins (two or more peptides) were identified in the 11-species biofilm and the 10-species biofilm, respectively, with corresponding false discovery rates (FDR) of 1.5% and 1.3%. The protein detection overlaps (i.e. similar proteins detected) between these two biofilm groups is shown in Fig. 2. In brief, 80.29% of the identified proteins appeared in both biofilm groups. The species-specific taxonomy of the identified protein numbers are provided in Table 2, whereas S1 Table presents the detailed information of total unique peptide counts and the annotations for each identified protein. The greatest overlap of proteins between the two kinds of biofilm was from *Fusobacterium nucleatum* (832 proteins). Interestingly, no uniquely identified proteins were detected for *P*. *intermedia*, in the absence of *A*. *actinomycetemcomitans* from the biofilm, while in its presence 117 proteins were uniquely identified. Accordingly, 95 proteins of *P*. *gingivalis* were uniquely identified in the 10-species biofilm lacking *A*. *actinomycetemcomitans*, while only 3 were uniquely identified in its presence in the 11-species biofilm. Of note, less than 10 proteins were identified for *A*. *oris* and *T*. *forsythia*, and less than 30 proteins for *T*. *denticola* in either of the biofilms, indicating that these species may be underrepresented in the corresponding databases. It is less likely that the proteins are underrepresented in the biofilm, as *T*. *forsythia* is numerically present at high levels in both biofilms (Table 1).

**Table 2:** Number of uniquely identified and overlapping proteins per species in two biofilm groups.

Species	11-species	Overlap	10-species
*A*. *actinomycetemcomitans*	97	13	1
*A*. *oris*	1	5	3
*F*. *nucleatum*	5	256	105
*C*. *rectus*	22	832	43
*P*. *gingivalis*	3	43	95
*P*. *intermedia*	117	299	0
*S*. *anginosus*	2	268	98
*S*. *oralis*	4	417	26
*T*. *denticola*	2	4	1
*T*. *forsythia*	5	19	4
*V*. *dispar*	31	632	2
*H*. *sapiens*	7	141	45

### *Aggregatibacter actinomycetemcomitans* alters the protein abundance of other species present in the biofilm

**Fig 3:**
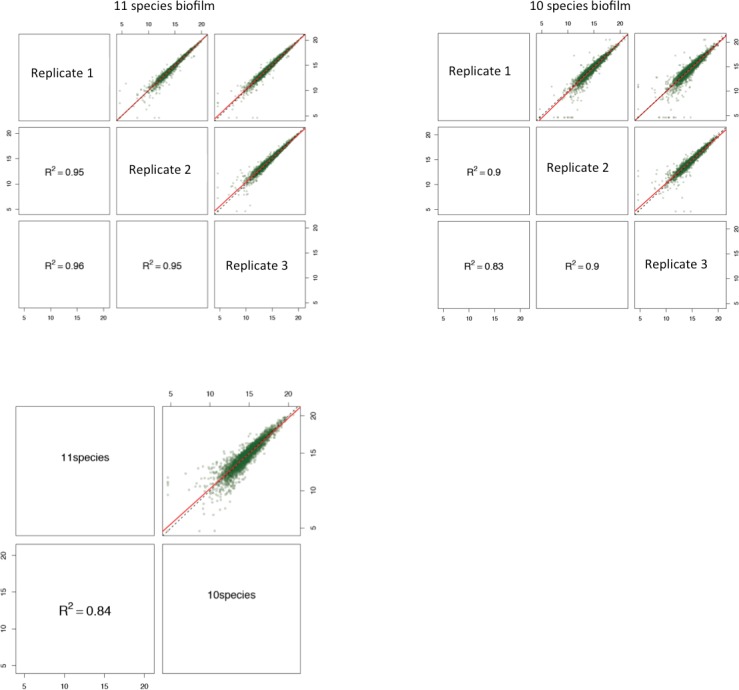
Quality control of the label-free quantitation data. Squared Pearson correlation coefficients (R^2^) of integrated peptide feature intensities are displayed for the comparisons within biological triplicates per biofilm group, and between the two biofilm groups. The linear regressions of the integrated peptide feature intensities of different conditions are indicated in red, whereas the dashed lines correspond to direct proportionality.

Label free quantification was used to analyse protein expressions between 11-species biofilm and its 10-species variant, lacking *A*. *actinomycetemcomitans*). A true regulation was considered when there was more than 2-fold difference in the levels of a given protein between the two groups, with *p*<0.05. Reproducibility between biological triplicates was evaluated using squared Pearson correlation coefficients (R^2^) of integrated peptide feature intensities. The R^2^ ranged between 0.95–0.96 in the 11 species biofilm group, and between 0.83–0.9 in the 10 species biofilm group, whereas this value was 0.84 between these two kinds of biofilm groups (Fig. 3).

A total of 790 proteins were identified, 278 of these were up-regulated in the 11-species biofilm compared with the 10-species biofilm, while 259 of these were down-regulated. The species-specific taxonomy of the numbers for identified proteins is provided in Table 3, whereas S2 Table presents the detailed information of the each corresponding protein. In brief, with the exception of *A*. *actinomycetemcomitans*, most of the up-regulated proteins in the 11-species biofilm belonged to *Prevotella intermedia* and *Veillonella dispar*, On the contrary, most down-regulated proteins belonged to *Porphyromonas gingivalis*, *Campylobacter rectus* and *Streptococci anginosus*. Of note, no quantifiable proteins were available in the case of *T*. *forsythia*.

**Table 3:** Number of label-free quantified proteins per species between two biofilm groups.

Species	Up-regulated	Un-regulated	Down-regulated
*A*. *actinomycetemcomitans*	46	2	0
*A*. *oris*	0	0	1
*C*. *rectus*	0	67	84
*F*. *nucleatum*	5	25	9
*P*. *gingivalis*	1	2	53
*P*. *intermedia*	199	56	0
*S*. *anginosus*	0	33	96
*S*. *oralis*	5	4	3
*T*. *denticola*	2	0	0
*V*. *dispar*	19	58	6
*H*. *sapiens*	1	6	7

In comparing the 11-species versus the 10-species biofilm, the proteins are defined as up-regulated, un-regulated or down-regulated. A significant (*p*<0.05) difference of 2-fold in protein levels was defined as “regulation”.

### *Aggregatibacter actinomycetemcomitans* causes distinctive changes in the functional ontology of quantified biofilm proteins

**Fig 4:**
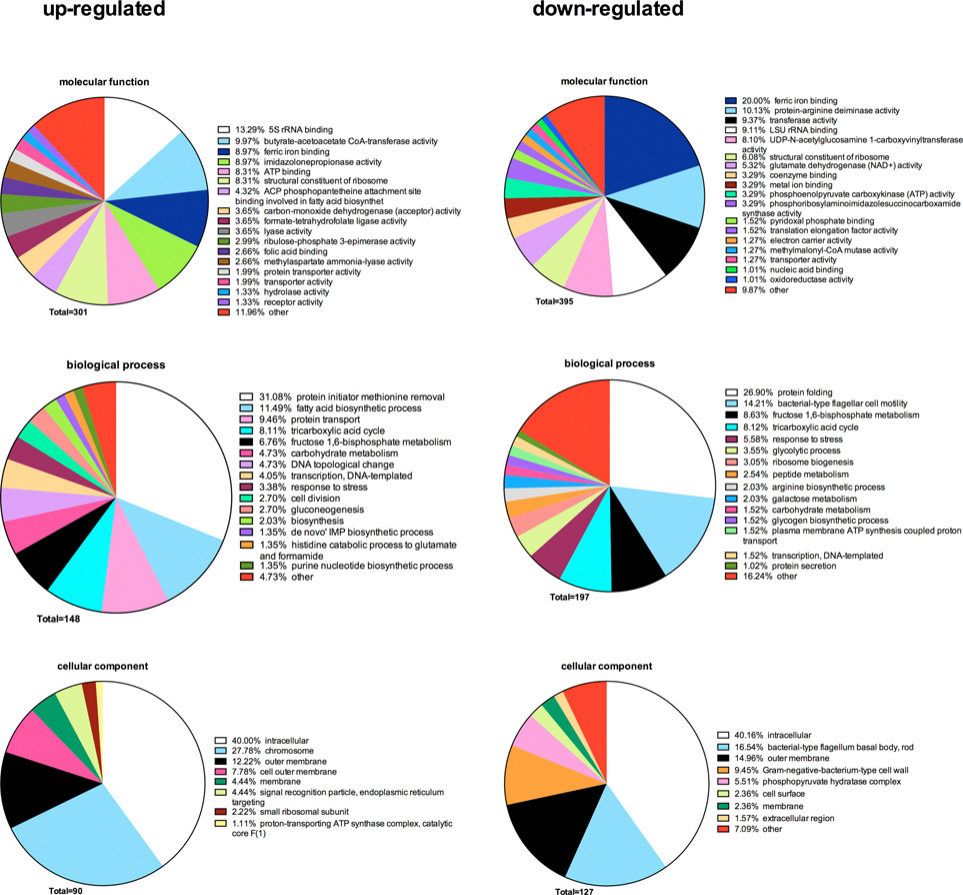
Annotation of overall regulated bacterial protein functions by enrichment of Gene Ontology (GO) terms. Based on the classifications of GO annotation, the overall bacterial functions were categorized into biological process, molecular function, and cellular component, and displayed in pie chart format. The numbers of GO terms for each of the three categories are shown, whereas the proportion of each specific subcategory is also provided. Subcategories with GO terms less than 1% are classified as “other”.

Following the label-free quantification, the functions of the regulated bacterial proteins were enriched according to Gene Ontology (GO) terms, with their redundant GO terms summarized and unified (Fig. 4). A total of 301, 148, and 90 GO terms for molecular function, biological process, and cellular component, respectively, were generated based upon the up-regulated proteins between 11-species biofilms and 10-species biofilms. On the contrary, based upon the down-regulated proteins, a total of 395, 197, and 127 GO terms were generated accordingly. Among GO terms of molecular function, “5S rRNA binding” (13.29%) was the most common function in up-regulated proteins in the presence of *A*. *actinomycetemcomitans* (11-species biofilm), while for down-regulated proteins, the most common function was “ferric iron binding” (20.00%), followed by “protein-arginine deiminase activity” (10.13%). Among GO terms of biological process, the most common up-regulated proteins belonged to “protein initiator methionine removal” (31.08%), while the most common down-regulated ones belonged to “protein folding” (26.90%). Regarding their cellular localization, more than 50% of up- and down-regulated proteins accounted for the intracellular/membrane-associated fraction.

## Discussion

With the increasing numbers of identified bacteria in oral biofilms, there are increasing needs in understanding their individual or communal functions. Consequently, contemporary research in the field focuses on the study of oral biofilm communities as a whole unit [13,21,33], rather than on individual bacterial species. Hence, by using proteomic approaches, this study aimed at elucidating the particular effects of *A*. *actinomycetemcomitans* in a multi-species *in vitro* bacterial community.

An elevated number of *A*. *actinomycetemcomitans* is associated with the aggressive form of periodontal disease in a longitudinal manner [23]. Thus, it is rational to postulate that, given its high specificity to aggressive periodontitis, this species may play an important role in orchestrating its microbial counterparts within a complex subgingival biofilm community. To this extent, previous experiments showed that it could integrate into a 6-species *in vitro* oral biofilm without dramatically changing the proportions of the other bacteria [34]. This was also the case in the present 11-species “subgingival” biofilm in experimental model, as it did not significantly affect the numbers of the other species present.

Interestingly, however, trends of protein regulations within the biofilm were distinct between individual constituent species. In the presence of *A*. *actinomycetemcomitans*, all regulated proteins of *P*. *intermedia* were found to be up-regulated, while all the regulated proteins of *C*. *rectus* and *S*. *anginosus* were found to be down-regulated. In the case of *P*. *gingivalis*, the vast majority of the quantified proteins were reduced in the presence of *A*. *actinomycetemcomitans*. Accordingly, both identification and label-free quantification data in this work indicate that *A*. *actinomycetemcomitans* regulates protein expression of other species present in the biofilm, although it may not directly reflect changes in bacterial numbers. These findings are perhaps in line with a recent report showing whole cell proteomic interactions between *P*. *gingivalis* and *F*. *nucleatum*: despite the close proximity in absolute numbers, qualitative changes were observed in protein composition, including reductions in proteins associated with amino-acid fermentation, glycolysis, translation, and biosynthesis of lipopolysaccharide as well as cell wall [29].

Both *P*. *intermedia* and *P*. *gingivalis* are black-pigmented bacteroides. Yet, in the present study, *A*. *actinomycetemcomitans* appears to have a differential effect in their proteomic biofilm interactions, causing an increase of all detected proteins in *P*. *intermedia* and decrease in almost all proteins in *P*. *gingivalis*. This finding could be of clinical relevance, as both *P*. *intermedia* and *A*. *actinomycetemcomitans* display a significant association with periodontal tissue breakdown [35]. *P*. *gingivalis* and *A*. *actinomycetemcomitans* can be isolated from different hosts, despite that both bacteria strongly associate with periodontal disease [36,37], and can in fact trigger distinctive pathogenic pathways [38]. Still, *P*. *gingivalis* displays aggregation properties with *A*. *actinomycetemcomitans* [39], and shows a competitive advantage over [40], or a mutualistic relationship with the latter [25]. Since aggregation between many bacteria used in our model are shown in dual species co-cultures [9], it is well possible that *A*. *actinomycetemcomitans* might not directly cause the differential regulation of proteins observed in the biofilm by its presence, but could rather work as a keystone bacterium that orchestrates the interaction between other constituted species. Such a role has already been described for and attributed to *P*. *gingivalis* [27,41,42].

As oral biofilms are polymicrobial and dynamic [1,43], both the genomic and proteomic profiles of the involved bacterial species are expressed differently from those in planktonic state [44]. Therefore, it makes more sense to consider the nature of expressed proteins, rather than numbers of each bacterial species in a biofilm. Interspecies signals should also be taken into count. For instance, autoinducer-2 (AI-2) produced by *S*. *oralis* is essential for the nutrition of *A*. *oris* [45], and AI-2 receptors were also found on *A*. *actinomycetemcomitans* [46]. To investigate the overall functions of the biofilm, the interpretation of all the regulated proteins should be evaluated as a whole rather than studied separately [47]. For this purpose, the protein profiles of the two forms of biofilm variants used in this study were pooled together in pursuing their regulated functions, including potential regulatory proteins for quorum sensing and mobile genetic elements. Different conjugative transposons are widespread in oral bacteria, including streptococci, *Veillonella* sp., *P*. *gingivalis*, *A*. *actinomycetemcomitans*, and *F*. *nucleatum* [48], and even horizontal gene transfer is common to oral biofilms [49]. Hence, the database used in this study contained all PubMed-retrieved protein information from each species, in order to avoid lost protein annotation. According to Uniprot, a universal protein resource with protein data created by combining the Swiss-Prot, TrEMBL and PIR-PSD databases, the final list of label-free quantified proteins comprised 96.4% of un-reviewed proteins, which are normally not accepted in most online functional annotation tools. Consequently, to give a general overview of the whole biofilm proteome in this case, we manually enriched all the GO terms for the label-free quantified proteins with Reduce + Visualize Gene Ontology (REVIGO) software, following the neighbour-joining method [50].

Based on the structured terminology of GO itself, all functions were divided into three separate ontologies: a) molecular function, b) biological process, and c) cell component. Only 3 out of 33 regulated GO molecular functions from label-free quantified proteins were enriched in both biofilms, which indicated that *A*. *actinomycetemcomitans* might have distinct effects on different molecular functions of the biofilm in general. Ferric iron binding, the most common down-regulated molecular function in the present *A*. *actinomycetemcomitans*-containing biofilm, was also as the fourth most common up-regulated molecular function, indicating a complex regulation among proteins of this category. Interestingly, regulation of ferric iron binding proteins has also been observed previously within a 3-species biofilm model [51]. This regulatory trend may not be surprising, as in the closed environment of the periodontal pocket, subgingival bacteria (including the ones used in this study) could utilize alternative, yet equally effective, iron-acquiring mechanisms in order to digest the host iron-containing proteins. For example, *A*. *actinomycetemcomitans* binds to lactoferrin and haemoglobin [52], *T*. *denticola* develops outer membrane protein HbpA with hemin binding ability [53], *P*. *gingivalis* employs specific outer membrane receptors, proteases, and lipoproteins for iron acquiring [54,55], and regulates the respective host cells responses [56]. Of note, gingipains, ferric iron binding proteases of *P*. *gingivalis*, including arginine-specific cysteine proteinase and lysine-specific cysteine proteinase, are also considered as virulence factors except for their hemin digestion ability [37]. Both gingipains were indeed found in Scaffold identification in the present study, with more peptides identified in the 10-species biofilm lacking *A*. *actinomycetemcomitans*. Hence, in the presence of this species, *P*. *gingivalis* gingipains may become more redundant for the entire biofilm community, as other factors of *A*. *actinomycetemcomitans* may also compensate for their iron-acquisition functions. As such, leukotoxin, a virulence factor of *A*. *actinomycetemcomitans*, is not only regulated in the presence of iron, but may also be involved in ferric iron acquisition [57]. Consequently, the overall turmoil of ferric iron-binding protein regulation within the biofilm might also affect the overall virulence of the biofilm towards the host tissue.

Apart from its involvement in ferric iron-binding related proteins, the present findings show that *A*. *actinomycetemcomitans* regulated the metabolic rate within the biofilm. The most common up-regulated molecular function in the 11-species biofilm, compared to its 10-species variant, was that of 5S RNA binding. This enriched protein function of structural ribosomal constituents and the fact that more proteins were identified from the small ribosome subunit (in cell component category), could easily be interpreted as an increase of bacterial growth [58]; however, given the fact that although the biofilms are cultured under stable growth conditions [59] they do not display differences in bacterial numbers irrespective of the presence of *A*. *actinomycetemcomitans*, increased bacterial growth within the 11-species biofilm is an unlikely explanation for this observation. The increase of the ribosome content is rather explained by increased protein transport, fatty acid biosynthetic process, and protein initator methoionine removal, as also observed in the up-regulated biological process category. Indeed, around 3% more GO terms responsible for cell division were identified, but this might be counter-balanced by deceased ribosome biogenesis and protein folding processes.

An altered metabolic rate was observed in an earlier study in a 3-species biofilm model [29,60,61], a trend that was also shown in this experimental model. In the presence of *A*. *actinomycetemcomitans* in the biofilm, biological processes like tricarboxylic acid cycle, fructose 1,6-bisphophate metabolism, and carbohydrate metabolism were enriched. By the present approach, it is not quite feasible to attribute these proteomic changes to one or another individual species, so at this stage they would have to be considered as a universal biofilm shift. Of note, fructose 1,6-bisphophate metabolism and carbohydrate metabolism were 2 of the 5 biological processes shared between these two kinds of biofilm variants (i.e. with or without *A*. *actinomycetemcomitans*). These two GO entities, together with glycolytic process, galactose metabolism, and arginine biosynthetic process, were enriched in absence of *A*. *actinomycetemcomitans*, indicating a strong alternation in the metabolic pathways of the biofilm. This may not be surprising, as for example, *A*. *actinomycetemcomitans* may utilize lactate from streptococci as energy source [15,62]. On the other hand, many glucose transports in *A*. *actinomycetemcomitans* can also be inhibited, as consequence of using lactate as carbon source [62]. These inhibited processes include a phosphoenolpyruvate (PEP): carbohydrate phosphotransferase system (PTS) [62], a bacterial unique system for concomitant transport and phosphorylation of carbohydrates in many species [63,64]. Among all our identified proteins, 2, 4, 12, 7, and 1 PTS proteins were identified from *A*. *actinomycetemcomitans*, *F*. *nucleatum*, *S*. *anginosus*, *S*. *oralis*, *V*. *dispar*, respectively, while based on the label-free data, all the non-*A*. *actinomycetemcomitans* PST proteins were identified as *S*. *anginosus*-derived, with 1 un-regulated and 4 down-regulated proteins. Regulation of these proteins definitely affected the output of the related GO function categories. Apart from direct effects of the PST regulation, other driving forces of selecting different organisms as consequence of *A*. *actinomycetemcomitans* utilizing lactate from streptococci, including shifts in pH (as an effect of lactate digestion) [65] and regulating quorum sensing factor AI-2 by PTS [66], may also contribute in biofilm formation and growth. In the context of a multiple species biofilm, such as the one used in this study, all the interactions between species become very complex and intricate. Therefore, understanding these interactions as a whole unit is not only more biologically reliable, but also a more efficient way to start deciphering biologically meaningful explanations.

The cellular localization category analysis for the most up- or down- regulated proteins delivered fractions associated with the intracellular/membrane component, with more than half of the identified GO terms falling into these categories. This indicated that *A*. *actinomycetemcomitans* could have a strong effect on mobilization of proteins in the bacterial cell compartments within the biofilm. Since by the present experimental approach we did not investigate the secreted protein fractions of the biofilm, it has not been possible to evaluate the overall turnover of extracellular proteins in the biofilm.

Elucidating the roles of specific bacterial species in a multiple species biofilm is a hard but eventually necessary task in understanding a biofilm as a community. The updated proteomic technologies provide powerful tools to understanding biofilms in a more detailed manner than earlier approaches [29,51,60,61]. Rather than identifying a panacea for the control of oral biofilms, the present study revealed shifts in the proteomic composition and functions within a complex *in vitro* biofilm environment, particularly focusing on the ecological pressures exerted by *A*. *actinomycetemcomitans* in the remainders of the bacterial community. On a further step, this type of analysis might be more meaningful if combined with a specific biological question (e.g whether ferric iron-binding-related functions are affected by *A*. *actinomycetemcomitans*), or in a co-culture system with host tissues, where one can more clearly predict the specific bacterial proteins in relation to their healthy or deleterious impact on the human host.

## Materials and Methods

### *In vitro* biofilm formation and harvesting

The 11-species biofilm used in this study included the following species: *Prevotella intermedia* ATCC 25611T (OMZ 278), *Aggregatibacter actinomycetemcomitans* JP2 (OMZ 295), *Campylobacter rectus* (OMZ 398), *Veillonella dispar* ATCC 17748T (OMZ 493), *Fusobacterium nucleatum subsp*. *nucleatum* (OMZ 598), *Streptococcus oralis* SK248 (OMZ 607), *Treponema denticola* ATCC 35405T (OMZ 661), *Actinomyces oris* (OMZ 745), *Streptococcus anginosus* ATCC 9895 (OMZ 871), *Tannerella forsythia* (OMZ 1047) and *Porphyromonas gingivalis* W50 (OMZ 308) was established. In parallel, the 10-species variant of this biofilm, lacking *A*. *actinomycetemcomitans* was also generated. The biofilms were grown in 24-well polystyrene cell culture plates on hydroxyapatite discs (diameter 13 mm). Briefly, 200 μl of bacterial cell suspensions containing equal densities (OD_550_ = 1.0) of each strain were mixed with 1.6 ml of growth medium consisting 60% saliva [67], 10% human serum, 30% modified fluid universal medium (mFUM) and 0.5% hemin to initiate biofilm formation. mFUM is a well-established tryptone-yeast-based broth medium designated as FUM [68] and modified by supplementing 67 mM Sørensen’s buffer (final pH 7.2). After 16 h of incubation in anaerobic conditions, additional 40 μl of *T*. *denticola* (OD_550_ = 1.0) were added to each well. The discs were further incubated 48 h until the biofilm was ready to be harvested (i.e total 64 h). During this period, the hydroxyapatite discs on which the biofilms were grown were dip-washed in 0.9% w/v of NaCl at 16 h, 20 h, 24 h, 40 h, 44 h, 48 h and 64 h, with the medium replenished at 16 h and 40 h, respectively.

For image analysis, the discs were put in wells containing 4% paraformaldehyde for at least 60 min for the fixation of the biofilms. For further analyses the rest of the biofilms was collected by vigorous vortexing for 3 min with 1 ml 0.9% w/v of NaCl, and then sonicated at 25 W for 5 seconds to obtain fine suspensions. These were then stored at -20°C before being processed for further analysis.

### Confocal laser scanning microscopy and image analysis

The localization pattern of *A*. *actinomycetemcomitans* within the biofilm structure was evaluated by CLSM. Briefly, the biofilm-containing discs stained by fluorescence *in situ* hybridization (FISH) using the Cy3-labelled *A*. *actinomycetemcomitans* 16S rRNA oligonucleotide probe Act639 (sequence from 5′ to 3′: CTCCAGACCCCCAGTATG, formamide concentration: 40%, Nacl concentration in wash buffer: 46mM) [34] and counter stained with YoPro-1 iodide and Sytox Green following the protocol described before [13]. A Leica SP-5 microscope (Center of Microscopy and Image Analysis of the University of Zürich), with a resonant scanner system (8000 Hz), an argon laser (458 nm/476 nm/488 nm/496 nm/514 nm excitation), and a helium neon laser (561 nm/594 nm/633 nm excitation) was used for visualization. Filters were set to 500–540 nm and to 570–630 nm, for the detection of green colour from YoPro-1 iodide & Sytox Green mixture and Cy3, respectively. All images were captured using a 63 × objective (glycerol immersion, NA 1.3). Stacked images were further processed using the Imaris 7.4.0 software (Bitplane), in order to virtually reconstruct the structure of the biofilm.

### Biofilm species quantification by quantitative real-time polymerase chain reaction (qPCR)

For individual species quantification in the biofilm, DNA was extracted from the bacterial suspensions using the GenElute bacterial genomic DNA kit (Sigma-Aldrich), as described before (Bao et al Virulence 2015). The qPCR assay was performed using SYBR Green PCR Master Mix (Life Technologies) in a StepOnePlus Real-Time PCR System (Applied Biosystems) with primers were designed using online NCBI/ Primer-BLAST tool (http://www.ncbi.nlm.nih.gov/tools/primer-blast), targeting the species-specific 16S rRNA gene (S3 Table). The numbers of each species were calculated on standard curves that were generated using extracted bacterial DNA of the corresponding planktonic cultures, and the theoretical genome weight of each organism from each strain according to the NCBI database as previous described [19].

### Protein extraction from biofilm pellet

The proteomic analysis was performed on the biofilm bacterial cell lysates from both biofilm variants, each represented in three biological triplicates. For this purpose, biofilm pellets were collected by centrifugation at 14,000 g for 15 min at room temperature, suspended with 30 μl of lysis buffer containing 4% w/v Sodium Dodecyl Sulfate (SDS), 0.1 mM dithiothreitol and 100 mM Tris-HCl pH 8.2, and incubated for 5 min at 95°C. High intensity focused ultrasound (UTR2000, Hielscher) was performed to lyse the bacterial pellets with 3 cycles of 3 min each, 0.5 cycle for intervals, and 65% ultrasonic amplitude. To avoid over-heating of the material, the samples were kept in an ice bath during the ultrasonic process and in wet ice for 3 min after each ultra-sonication cycle.

### Protein digestion and C18 clean up

After lysis, the protein concentrations from both biofilm variants, each represented by biological triplicates, were measured with Qubit Protein Assay Kit (Life Technologies). Then, 20 μg of extracted bacterial protein were subjected to ultrafiltration for filter device with relative molecular mass (Mr) cut-offs of 30,000 (30k filter) to efficiently retain proteins and allow removal of impurities following the similar protocol of Wisniewski et al [69] with minor modifications described below. Briefly, 200 μl of 8M urea in 100 mM Tris/HCl buffer (pH 8.2) were added and the samples were centrifuged at 14,000g for 20 min at 35°C. This step was repeated once. Then 100 μl of urea buffer with additional 0.05 M iodacetamide was added to the filters and centrifuged at 14,000 g for 20 min at 35°C. Filters were washed three times with 100 μl urea buffer and followed by two washes with 100 μl of 0.5 M NaCl at 14,000g for 17 min at 35°C. Proteins were digested in 120 μl of 0.05M Triethylammoniumbicarbonat (TEAB) using trypsin (Promega) at enzyme to protein ratio of 1:40 in wet-cell chamber overnight. The peptides were collected by centrifugation at 14,000g for 20 min. Digestion was stopped by adding trifluoroacetic acid (TFA) to a final concentration of 0.5% and diluted with 400ul 3% acetonitrile in 0.1% TFA to optimal the binding volume. Peptide mixtures were desalted using reverse phase cartridges Finisterre SPE C18 (Wicom International AG) according to the following procedure: cartridge was wet with 1 ml of 100% methanol, washed with 1 ml 60% of acetonitrile (ACN) in 0.1% TFA, equilibrated with 2 ml of 3% ACN in 0.1% TFA, load-acidified digested, washed with 6 ml of 3% ACN in 0.1% TFA, and eluted with 0.5 ml of 60% ACN in 0.1% TFA. Peptides were dried using a vacuum centrifuge, resolubilized with 30 μl 3% ACN in 0.1% formic acid, and frozen at -20°C, until further use.

### LC-MS/MS analysis and database search

Tryptic peptides of obtained from the biofilm lysates were analyzed on a Q-Exactive mass spectrometer (Thermo Fisher Scientific). Chromatographic separation of peptides was performed on an Easy nano-flow HPLC system (Thermo Fisher Scientific) coupled to a 15 cm fused silica emitter, 75 μm diameter, packed with a ReproSil-Pur C18-AQ 120 A and 1.9 μm resin (Dr. Maisch HPLC GmbH). Peptides were loaded on the column and separated with a linear gradient of acetonitrile/water, containing 0.1% formic acid, at a flow rate of 300 nl/min. A gradient from 2 to 35% acetonitrile in 120 minutes was used. Mass spectra were acquired in a data-dependent manner, with an automatic switch between MS and MS/MS using a top 12 method. MS spectra were acquired in the Orbitrap analyzer with a mass range of 300–1700 *m*/*z*. Higher energy collisional dissociation (HCD) peptide fragments, acquired at 28 normalized collision energy, were analyzed at high resolution in the Orbitrap.

### Database search analysis and label-free quantification

Each file was searched with Mascot (version 2.4.1) against a database (containing 634157 sequence, 207,052,936 residues) consisting of *Homo sapiens* database (including isoforms) from Uniprot (release date 22 May 2014, containing 88,708 forward sequences and 88,708 reverse sequence as decoy), all protein lists the 11 bacterial species from NCBI database (release date 28 February 2014, containing 228,240 forward sequences and 228,240 reverse sequence as decoy), and a contaminates database with 261 sequences. The precursor ion tolerance was set to ±10 ppm and a fragment-ion mass tolerance of ± 0.05 Da. For the search criteria, tryptic digests were allowed, up to 2 missed cleavages per peptide, carbamidomethylation (C) as a fixed modification on cysteine, and oxidation (M) as variable modification on methionine residues.

For the protein identification, the Mascot research results of the biofilms were imported into Scaffold (version Scaffold_4.2.1, Proteome software) to validate MS/MS-based peptide and protein identifications with protein threshold: 3.0% FDR for protein threshold, 2 minimal peptides, and 1.0% FDR for peptide threshold.

For the label-free quantification, the Mascot search results were imported back to Progenesis LC—MS software (Nonlinear Dynamics) for feature detection, alignment, and quantification. The proteins identified by similar sets of peptides were grouped and only non-conflicting peptides with specific sequences for single proteins in the database were employed for protein quantification. All the exported files were further processed using Mascot, Scaffold, and imported back to Progenesis before loading the final protein reports to SafeQuant (v.1.0.1) for the statistical validation. The statistical significance of each ratio was given by its *q*-value (*q*< 0.05 significance level), obtained by calculating modified t-statistic *P* values [70] and adjusting for multiple testing [71].

### Ontology analysis

The lists of regulated protein identifications (Uniprot IDs) from SafeQuant, with *q* values less than 0.05 and log_2_ ratios more than 1 fold or less than-1 fold, were used to estimate the differences of the presence of *A*. *actinomycetemcomitans* in the biofilm. Human proteins present in the experimental system (potentially deriving from the serum growth supplement or the pellicle) were excluded from further analyses. Only proteins of the relevant 11 bacterial species constituting the biofilms were imported into the Uniprot (release date 10 October 2014) to generate lists of Gene Ontology (GO) function using Retrieve/ID Mapping function. The similar GO terms generated from Uniprot were enriched by REVIGO (release date 13 October 2014) [72] with the medium allowing similarity of 0.7 to replace their original terms in the lists. Then, functions of the proteins were manually summarized in pie charts based on their GO terms. GO terms that less than one percentage were clustered into category “other”.

### Statistical evaluation

All data present in the experiment derives from triplicate biofilm cultures. For the bacterial determination by qPCR, the values were logarithmically transformed, and then inserted to Prism v.6 software (GraphPad, La Jolla California USA). The statistical differences (*P* ≤ 0.01) between the groups were calculated by student t-test.

## Supporting Information

S1 Table List of identified proteins from Scaffold. Accession numbers, identified protein names, taxonomy, peptide count from each replicate, and overlap in different groups of identified proteins from 11-species biofilms or 10-species biofilms are presented in the table. The proteins were listed following the similarity between the proteins given by Scaffold.
(XLSX)

S2 Table List of label-free quantified proteins from Progenesis. Accession numbers, identified protein names, taxonomy, way of regulation, log_2_ ration, *q*-value, and peptide used for quantification of identified proteins from 11-species biofilms or 10-species biofilms are presented in the table. Proteins with log_2_ ratio more than 1-fold or less than 1-fold with *q*< 0.05 were considered as regulated proteins.
(XLSX)

S3 Table Primer sequences and related information. (DOCX)
