# Supplementary material for: Quantitative Proteomics Reveal Distinct Protein Regulations Caused by Aggregatibacter actinomycetemcomitans within Subgingival Biofilms
Source: PLoS One. 2015 Mar 10;10(3):e0119222. doi: 10.1371/journal.pone.0119222 (PMC4355292; doi:10.1371/journal.pone.0119222)
Supplement: S3 Table — (DOCX) [file pone.0119222.s003.docx]

**S3 Table**: **Primer sequences and related information**

| **Organism** | **Sequence (5′**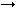**3′)** | **Strand on template** | **Size of amplicon (bases)** | **T_m_ (°C)** | **Ref #** |
| --- | --- | --- | --- | --- | --- |
| *Streptococcus anginosus* | ACCAGGTCTTGACATCCCGATGCTA | + | 76 | 59.25 | [[19](#_ENREF_19)] |
|  | CCATGCACCACCTGTCACCGA | − |  | 59.04 |  |
| *Streptococcus oralis* | ACCAGGTCTTGACATCCCTCTGACC | + | 70 | 59.42 | [[19](#_ENREF_19)] |
|  | ACCACCTGTCACCTCTGTCCCG | − |  | 59.85 |  |
| *Actinomyces oris* | GCCTGTCCCTTTGTGGGTGGG | + | 71 | 59.57 | [[19](#_ENREF_19)] |
|  | GCGGCTGCTGGCACGTAGTT | − |  | 60.32 |  |
| *Veillonella dispar* | CCCGGGCCTTGTACACACCG | + | 62 | 59.7 | [[19](#_ENREF_19)] |
|  | CCCACCGGCTTTGGGCACTT | − |  | 59.83 |  |
| *Fusobacterium nucleatum* | CGCCCGTCACACCACGAGA | + | 75 | 59.04 | [[19](#_ENREF_19)] |
|  | ACACCCTCGGAACATCCCTCCTTAC | − |  | 59.48 |  |
| *Campylobacter rectus* | TCACCGCCCGTCACACCATG | + | 57 | 59.35 | [[19](#_ENREF_19)] |
|  | CCGGTTTGGTATTTGGGCTTCGAGT | − |  | 59.5 |  |
| *Prevotella intermedia* | GCGTGCAGATTGACGGCCCTAT | + | 68 | 59.61 | [[19](#_ENREF_19)] |
|  | GGCACACGTGCCCGCTTTACT | − |  | 60.24 |  |
| *Porphyromonas gingivalis* | GCGAGAGCCTGAACCAGCCA | + | 90 | 59.07 | [[19](#_ENREF_19)] |
|  | ACTCGTATCGCCCGTTATTCCCGTA | − |  | 59.44 |  |
| *Treponema denticola* | TAAGGGACAGCTTGCTCACCCCTA | + | 55 | 58.84 | [[19](#_ENREF_19)] |
|  | CACCCACGCGTTACTCACCAGTC | − |  | 59.76 |  |
| *Tannerella forsythia* | CGATGATACGCGAGGAACCTTACCC | + | 72 | 59.07 | [[19](#_ENREF_19)] |
|  | CCGAAGGGAAGAAAGCTCTCACTCT | − |  | 58.01 |  |
| *Aggregatibacter actinomycetemcomitans* | GTGGGGAGCAAACAGGATTAG | + | 260 | 58.63 | [[22](#_ENREF_22)] |
|  | CCTAAGGCACAAACCCATCTC | − |  | 58.63 |  |
| T_m_, melting temperature | | | | | |
